# Real-Time Adaptive Motion Management in Prostate Stereotactic Body Radiation Therapy: Clinical and Dosimetric Analysis of 25 Patients

**DOI:** 10.7759/cureus.82197

**Published:** 2025-04-13

**Authors:** Lee C Goddard, Jonathan Cabrera, Justin Tang, Madhur K Garg, Wolfgang A Tome

**Affiliations:** 1 Radiation Oncology, Montefiore Medical Center, Bronx, USA; 2 Institute for Onco-Physics, Albert Einstein College of Medicine, Bronx, USA

**Keywords:** adaptive radiation therapy, fiducial tracking, prostate motion, prostate sbrt, radixact synchrony

## Abstract

Purpose: Stereotactic body radiation therapy (SBRT) is a well-established treatment for localized prostate cancer, offering excellent tumor control with reduced treatment fractions. However, prostate motion due to physiological processes poses a challenge to precise dose delivery. This study evaluates the dosimetric outcomes, prostate-specific antigen (PSA) response, and toxicity profile of prostate SBRT using real-time adaptive motion management with the Radixact Synchrony system (Accuray Inc., Madison, WI).

Methods: A retrospective analysis was conducted on 25 prostate cancer patients treated with SBRT (40 Gy in 5 fractions) using the Radixact Synchrony system between January 2021 and December 2023. All patients underwent CT simulation with fiducial markers, and 24/25 patients had MRI fusion for target and organ-at-risk (OAR) delineation. Treatment plans were generated using either the “Classic” or “VOLO Ultra” algorithms. Intrafraction prostate motion was analyzed, and toxicity was assessed using the Common Terminology Criteria for Adverse Events (CTCAE) v4.03 scale at regular follow-up intervals.

Results: The median clinical target volume (CTV) for the prostate was 46.8 cm³ (range, 22.6-107.3 cm³), and the median pre-treatment PSA was 6.52 ng/mL. All plans achieved favorable target coverage while adhering to OAR dose constraints. The median post-treatment PSA value was 0.30 ng/mL with a median follow-up time of 28 months, with all patients achieving PSA levels below 2.0 ng/mL at 15 months. Motion >2 mm was observed in at least one treatment fraction for all but one patient. Real-time adaptive motion management successfully corrected for prostate displacement, with an average imaging interval of 8.4 seconds. A total of 14 patients reported new grade 1 genitourinary (GU) symptoms, and three patients experienced grade 2 GU toxicity post-treatment.

Conclusion: Prostate SBRT with real-time adaptive motion management using the Radixact Synchrony system achieves excellent target coverage while mitigating the effects of intrafraction motion. The observed PSA response supports its efficacy, and OAR dose metrics remain within acceptable limits. While real-time motion adaptation was beneficial for nearly all patients, further studies with larger cohorts and patient-reported outcomes are warranted to assess long-term toxicity and quality of life.

## Introduction

Stereotactic body radiation therapy (SBRT) is an increasingly common treatment modality for localized prostate cancer [1]. Prostate SBRT offers excellent tumor control while minimizing organ-at-risk (OAR) toxicity and reducing the number of treatments, compared to conventional fractionation schedules [2]. Prostate motion due to physiological processes such as respiration, bladder filling, and rectal peristalsis presents a significant challenge in maintaining precise dose delivery necessary for SBRT [3-5]. Real-time adaptive motion management strategies aim to mitigate this uncertainty, enhancing treatment accuracy and potentially reducing toxicity to surrounding OARs.

The Radixact Synchrony system (Accuray Inc., Madison, WI) integrates helical tomotherapy with intra-fractional motion tracking, allowing real-time adaptation of beam delivery to target motion. "Quasi-static motion correction" requires at least one fiducial to be implanted within the prostate, although three to four are typically recommended. Kilo-voltage (kV) planar images are acquired during treatment delivery, and fiducial markers within the prostate are detected. The treatment jaw position and/or multi-leaf collimator (MLC) openings are offset, based on the detected fiducial position, with the offset being updated with each newly acquired kV image. Two to six planar images can be acquired per gantry rotation, with a minimum gantry period of 11.8 seconds and a maximum period of 60.0 seconds. This results in imaging periods of between 2.0 and 30.0 seconds, depending on the number of imaging angles selected and the planned gantry period.

In this study, we report on achievable treatment plan dose volume histogram (DVH) metrics, prostate-specific antigen (PSA) response, and physician-reported toxicities for prostate cancer patients treated with SBRT using real-time adaptive motion management.

## Materials and methods

This retrospective study includes 25 patients diagnosed with localized prostate cancer who received SBRT treatment between January 2021 and December 2023 using the Radixact Synchrony system for "quasi-static" adaptive real-time motion management. All patients were treated with definitive intent, receiving a total dose of 40 Gy in five fractions prescribed to the evaluation planning target volume (PTV Eval), as described below.

Each patient underwent a CT simulation in the supine position with a comfortably full bladder and an empty rectum. Patients were asked to repeat bladder filling and bowel emptying prior to treatment to optimize prostate localization and reduce inter-fraction prostate position variability. One patient was rescanned and treated in the prone position due to small bowel proximity to the planning target volume (PTV) in the supine position, which was improved in the prone position. Three fiducial markers were implanted in the prostate prior to simulation for all patients. A total of 23 patients had rectal spacer gel inserted at the time of fiducial insertion. The use of rectal spacer gel has been shown to improve rectal dosimetry by increasing the distance between the prostate gland and rectal wall [6]. Two patients who were unable to undergo rectal spacer gel insertion were simulated and treated with an air-filled endo-rectal balloon (ERB) with 150cc filled volume. ERBs have been shown to improve rectal dosimetry for prostate SBRT patients, hence their use in patients unable to undergo rectal spacer insertion [7].

MRI imaging was acquired in the treatment position for 24 patients immediately following CT acquisition. MRI fusion was performed based on fiducial positions for clinical target volume (CTV prostate) and OAR (neurovascular bundle and urethra) delineation when available. For one patient, unable to undergo MRI imaging, the CTV prostate was delineated on CT imaging and the urethra and neurovascular bundles were not contoured as OARs. CT imaging was used for delineation of the seminal vesicles (SV) and remaining OARs (bladder wall, bowel (small), colon (sigmoid), femoral heads, penile bulb, rectal wall and urogenital diaphragm). Bladder and rectal walls were generated using an initial 2 mm inner margin from the organ outer contour, this margin was increased based on individual patient anatomy as seen on CT and/or MRI imaging.

The clinical target volume (CTV) included the entire prostate, and in 20 patients, the proximal seminal vesicles (CTV SV), with the extent of CTV SV inclusion determined by the treating physician. The PTV was generated using a 5 mm margin from the CTV in all directions, except posteriorly, where a 3 mm margin was applied to reduce rectal dose. A PTV Eval was generated by cropping the PTV 5 mm from the small and large bowel, 2 mm from the rectal wall and urethra, and 1 mm from any remaining OARs. The prescription dose of 40 Gy was prescribed to cover at least 95% of the PTV Eval, V40 Gy ≥ 95%, and the minimum dose to the entire PTV prescribed to be 36.25 Gy, V36.25 Gy ≥ 99%. D_max_, defined as the dose to 0.03 cm^3^, should be less than 45 Gy, D_max_ ≤ 45 Gy, and the 42 Gy isodose line should not extend beyond the PTV. Treatment plans were created using the “Precision” treatment planning system (TPS), with 14 plans being generated using the “Classic” algorithm and eleven plans generated with the “VOLO Ultra” algorithm.

Toxicity data were collected at each follow-up appointment, per Common Terminology Criteria for Adverse Events (CTCAE) 4.03 grading scale. Patients were asked to return for a follow-up visit 30 days post-treatment, then at 3, 6 months and 12 months post-treatment and then every 6-12 months.

## Results

Table 1 shows the characteristics of the 25 patients included in this study. The median age at the time of treatment was 65 years (54-77 years). CTV prostate volumes ranged from 22.6 to 107.3 cm^3^ with a median value of 46.8 cm^3^. The median pre-treatment PSA value was 6.52 ng/mL (3.73-16.00 ng/mL). Table 2 shows the average achieved value for various OAR constraints as well as the range of achieved values and the clinical constraints. Table 3 shows the average and range of achieved target doses. All plans were generated using the 2.5 cm dynamic jaw. The average PTV conformality index was 1.13 (1.03-1.27), and the average homogeneity index, defined in the Precision TPS as the ratio of D_max_ to the prescription dose, was 1.09 (1.03-1.18). Average treatment time was 606 s (420-879 s) with an average gantry period of 34.1 s (19.0-57.4 s) and an average imaging interval of 8.4 s (2.6-18.3 s).

**Table 1:** Patient Characteristics Prostate-specific antigen (PSA)

Patient	Age at time of treatment (years)	Risk group	Gleason score	Stage	Pre-treatment PSA (ng/mL)	Prostate Volume (cm^3^)	Treatment Interval (days)
1	62	Intermediate	7 (3+4)	2B	3.7	31.9	10
2	58	Intermediate	6 (3+3)	2A	12.6	54.0	14
3	61	Low	6	1	6.5	52.5	13
4	73	Intermediate	7 (4+3)	2C	6.5	44.1	10
5	64	Intermediate	7 (3+4)	2B	5.3	35.9	13
6	65	Intermediate	7 (4+3)	2C	5.9	22.6	9
7	76	Intermediate	6 (3+3)	2A	10.3	107.3	9
8	74	Intermediate	7 (4+3)	2C	5.6	61.6	10
9	66	Intermediate	7 (3+4)	2B	5.6	27.5	11
10	69	Intermediate	6 (3+3)	2A	12.4	50.4	9
11	69	Intermediate	7 (3+4)	2B	6.8	70.7	9
12	63	Intermediate	7 (3+4)	2B	14.8	41.1	9
13	58	Intermediate	7 (3+4)	2B	5.1	30.1	9
14	63	Intermediate	6 (3+3)	2A	10.0	35.6	10
15	61	Low	6	1	5.4	29.8	10
16	54	Intermediate	7 (4+3)	2C	7.6	58.5	9
17	75	Intermediate	7 (3+4)	2C	9.2	35.2	9
18	77	Intermediate	6 (3+3)	2A	10.2	43.6	9
19	73	Low	6 (3+3)	1	8.5	54.9	9
20	72	Intermediate	7 (3+4)	2B	8.8	32.7	11
21	64	Intermediate	7 (3+4)	2B	3.9	46.9	9
22	56	Low	6 (3+3)	1	4.5	64.7	11
23	73	Intermediate	7 (3+4)	2B	16.0	46.6	11
24	65	Intermediate	7 (3+4)	2B	7.7	57.4	11
25	75	Intermediate	6 (3+3)	2A	13.0	32.3	9

**Table 2:** Mean achieved organ-at-risk dose metrics with the range of measured values shown in parentheses and the clinical constraint shown in bold.

	D_0.03 cm_^_3_^(Gy)	D_1 cm^3^_(Gy)	D_3 cm_^_3_^(Gy)	D_50%_ (Gy)	D_mean_ (Gy)
Bladder wall	41.2 (38.9-43.5) 45.0	40.1 (38.0-41.9) 42.0	-	11.4 (1.3-22.4) 24.0	-
Bowel (small)	4.7 (0.4-19.9) 25.0	-	-	-	-
Colon (sigmoid)	12.4 (1.7-24.8) 29.0	-	-	-	-
Femoral heads	16.1 (3.0-27.6) 31.0	-	-	-	-
Neurovascular bundle	41.9 (39.9-43.6) 45.0	-	-	-	-
Penile bulb	18.8 (2.0-36.3) 45.0	-	4.7 (1.7-12.8) 22.5	-	-
Prostatic urethra	37.8 (35.6-41.1) 42.8	-	-		36.1 (33.7-38.7) 38.0
Rectal wall	38.2 (31.8-41.4) 42.8	33.5 (27.9-36.4) 38.5	-	15.5 (7.9-23.4) 24.0	
Urogenital diaphragm	39.6 (7.2-43.4) 42.8	-	-	-	-

**Table 3:** Average target dose volume histogram metrics Clinical target volume (CTV), proximal seminal vesicles (SV), planning target volume (PTV).

	D_99%_ (Gy)	V_36.25Gy_ (%)	V_40Gy_ (%)	D_0.03cm^3^_ (Gy)
CTV Prostate	35.9 (32.6-40.0)	97.9 (91.9-100)	86.4 (74.1-99)	-
CTV SV	39.4 (32.1-42.0)	98.6 (79.2-100)	94.2 (49.1-100)	-
PTV	35.3 (28.4-37.9)	97.7 (88.6-100)	84.5 (65.5-95.2)	-
PTV Eval	38.6 (32.1-40.1)	-	95.6 (89.8-99.2)	43.1 (41.1-46.8)

**Figure 1:**
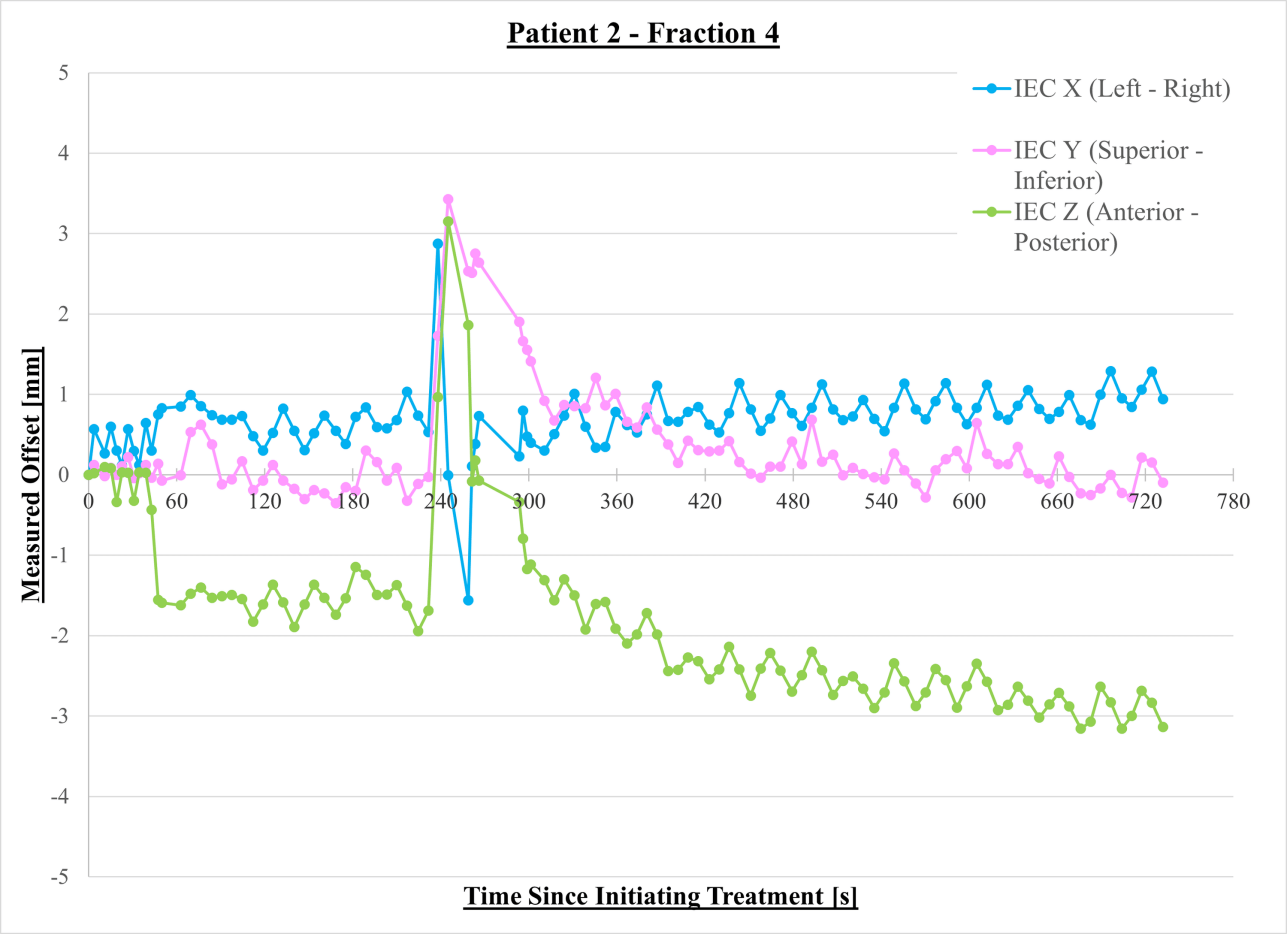
Measured target offsets for Patient 2, fraction 4

Figure 1 shows an example graph of measured target offsets vs. time for Patient 2, treatment fraction 4. There was a large motion at approximately 240 s after treatment was initiated, and a gradual drift can also be seen in the posterior direction. Table 4 shows the motions that were measured for each patient and treatment fraction. These motions are classified as: stable (S) - no motion > 2 mm, drifting (D) - gradual motion changes > 2 mm, abrupt small magnitude (As) - rapid motion changes < 5 mm, abrupt large magnitude (Al) - rapid motion changes > 5 mm. Real-time motion management could be employed for each patient for all treatment fractions. All patients, except one, were found to have at least one fraction where motions greater than 2 mm were measured in at least one direction. Patient 18, the patient who did not have any treatments where a motion > 2 mm was found, was treated with an ERB, which limits the prostate motion due to organ motion. Patient 21 was also treated with an ERB; however, for two fractions, he showed motions > 2mm, perhaps due to gross patient motions or large magnitude organ filling. Abrupt magnitude motions were measured 77 times over 36 treatments, these motions typically caused the treatment to halt. After interrupting treatment delivery, for the majority of treatments, a new model was successfully built and treatment resumed; however, for seven of these treatments, a repeat CT was performed and the patient position adjusted before continuing treatment. Repeat CTs and patient repositioning were performed mid-treatment if persistent large magnitude shifts were found. Due to the MLC width, MLC-based corrections are of a coarser resolution than jaw-based corrections. For example, if a shift of -3 mm was noted in the IEC X or IEC Z directions, the synchrony system will offset the delivery by one MLC leaf, resulting in the dose distribution being shifted to +3 mm. Large magnitude IEC Y offsets can result in the shifts beyond the mechanical capabilities of the jaw, and jaw offsets also result in the effective output being reduced. Hence, if either of these conditions were found, a mid-treatment CT was necessary.

**Table 4:** Measured Motion Type for each Treatment Delivery. Fraction (Fx). Stable (S) – no motion > 2 mm, drifting (D) – gradual motion changes > 2 mm, abrupt small magnitude (As) – rapid motion changes < 5 mm, abrupt large magnitude (Al) – rapid motion changes > 5 mm. * denotes patients treated with an endo-rectal balloon.

Pt	Fx 1	Fx 2	Fx 3	Fx 4	Fx 5
1	S	S	S	S	D
2	D	As	As	Al	D
3	D	S	As	D	S
4	Al	Al	Al	Al	Al
5	Al	Al	S	S	D
6	D	D	As	S	D
7	S	As	D	S	As
8	Al	D	Al	Al	As
9	S	As	S	As	Al
10	S	D	D	D	S
11	S	D	D	D	D
12	D	As	S	D	As
13	D	S	D	S	Al
14	D	S	S	As	S
15	S	As	Al	As	D
16	Al	As	S	Al	S
17	D	D	S	D	S
18*	S	S	S	S	S
19	As	D	As	Al	As
20	D	D	As	As	D
21*	S	S	S	As	D
22	S	S	As	S	As
23	As	S	As	D	D
24	D	As	Al	Al	Al
25	D	D	D	Al	As

The average number of kV planar images acquired per treatment delivery was 79 (56-73). Patient 1 was treated with a software version that did not record the imaging dose; this patient was excluded from the following imaging dose metrics. The average kV planar imaging dose was 0.6 cGy (0.3-2.1 cGy) per treatment delivery. A total of 23/24 patients received kilo-voltage helical fan beam CT (kVCT) volumetric imaging for patient setup; the average number of kVCTs acquired per treated fraction was 1.6 (1.0-2.8), which includes repeat kVCTs acquired before initiating treatment and kVCTs acquired mid-treatment. The average total CT dose index (CTDI) per patient treatment was 2.9 cGy (1.0-5.1 cGy). Patient 19 received mega-voltage helical fan beam CT (MVCT) volumetric imaging due to the presence of a hip prosthesis. This patient received one pre-treatment MVCT before each treatment, with a CTDI of 2.6 cGy per image.

**Figure 2:**
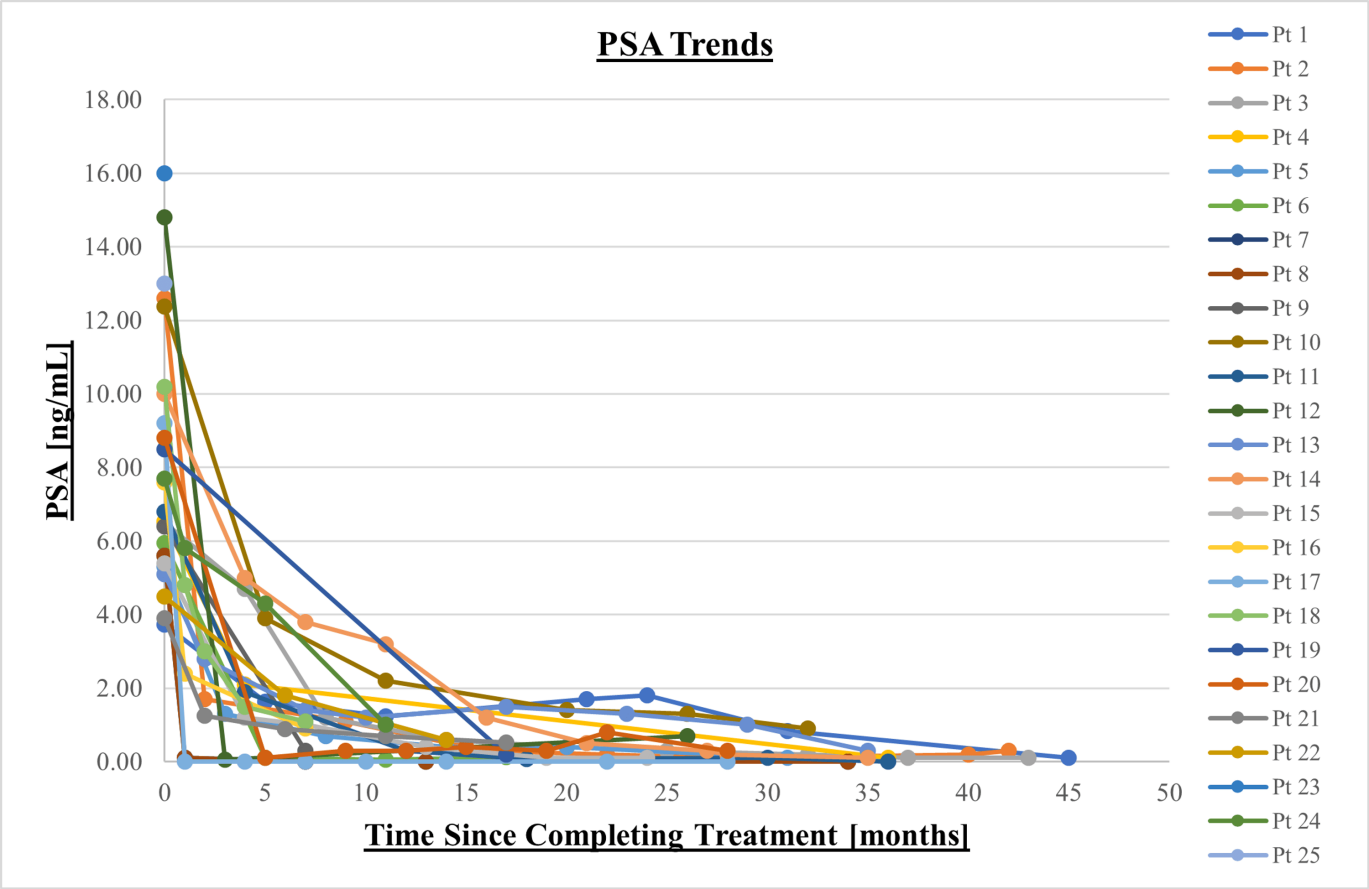
Measured PSA pre-treatment and at follow-up Prostate-specific antigen (PSA)

Figure 2 shows pre-treatment PSA levels for all patients with follow-up data, as well as PSA trends post-treatment. The median post-treatment PSA value was 0.30 ng/mL (0.10-1.10 ng/mL) with a median follow-up time of 28 months (6-45 months), with two patients being lost to follow-up. For all patients, PSA levels fell below 2 ng/mL at 15 months following completion of therapy. PSA levels continued to decrease, with three patients experiencing a PSA bounce at 22-26 months testing, but subsequently continuing to exhibit a monotonic decrease in PSA.

A total of fourteen patients reported new grade one genitourinary (GU) symptoms, and three patients reported grade two symptoms post-treatment. Five patients reported grade one GU symptoms, and three patients reported grade one fatigue post-treatment. 

## Discussion

Our study evaluates the achieved dosimetric metrics, PSA response, and toxicity profile of prostate SBRT delivered with the Radixact Synchrony system. The results demonstrate that the Radixact system can achieve favorable target coverage while maintaining OAR constraints within established limits.

The achieved PTV coverage aligns well with prior prostate SBRT studies, with V36.25Gy exceeding 97% on average. The use of rectal spacer gel in 23/25 patients likely contributed to the favorable rectal dose distributions.

The substantial decline in PSA levels post-treatment, with a median value of 0.30 ng/mL, underscores the efficacy of prostate SBRT with real-time motion adaptation. This is consistent with prior SBRT data, where durable biochemical control is observed in most patients. While the follow-up period for PSA control is short, it shows that the motion management is not leading to geographic misses. The follow-up period (median 28 months) is sufficient to assess early toxicity, but long-term follow-up is necessary to evaluate sustained biochemical control and late toxicity.

Intrafraction motion remains a critical challenge in prostate SBRT, as shown in our study. As can be seen from Table 4, while for the majority of patients a stable prostate position was found for some treatment fractions in their treatment course, all patients experienced either drifting and/or abrupt motion changes of the prostate during their course of therapy. A total of 6/25 patients had significant prostate motions for all treatment fractions, clearly indicating the value of real-time motion management. The Radixact Synchrony system mitigates the impact of motion on dose delivery uncertainty and allows for precise dose delivery, resulting in the use of PTV margins smaller than those utilized in this work [8]. Reducing PTV margins would also lead to more favorable OAR dose distributions than the values we present.

All but one patient benefited from the real-time adaptive motion management. The use of ERB may obviate the need for real-time management for one patient; however, in the other patient with ERB, the system detected a large enough variation to adjust treatment parameters. In addition, the use of a large air-filled ERB is also fairly uncomfortable for the patient and significantly adds to machine time. The average imaging interval of 8.4 seconds (2.6-18.3 s) enabled frequent positional updates, minimizing target displacement and potential dosimetric degradation.

## Conclusions

While this study provides valuable insights into prostate SBRT with Radixact Synchrony, it is limited by its retrospective design and relatively small sample size. Additionally, toxicity outcomes were based on physician-reported data rather than patient-reported measures, which may underrepresent symptom burden. Future studies should incorporate patient-reported outcomes and quality-of-life assessments to comprehensively evaluate treatment tolerability. 

Our findings suggest that prostate SBRT with Radixact Synchrony provides excellent target coverage while effectively managing intrafraction motion. The observed PSA response and OAR dose metrics support its use as a treatment modality for localized prostate cancer.

After the initial run-in phase our institution has adopted smaller PTV margins than those utilized in this study; however, insufficient time has passed to collect and analyze enough data to present definitive results on the impact of this change on OAR doses and toxicity outcomes. Future studies should allow for longer follow up periods, as well as comparisons between patient cohorts utilizing differing PTV margin, to further demonstrate the benefits of motion compensation in prostate SBRT.
